# Excision of a Distal Tibial Interosseous Osteochondroma Through Posterolateral Approach: A Case Report

**DOI:** 10.7759/cureus.59592

**Published:** 2024-05-03

**Authors:** Soumyadip Sen, Abheek Kar, Abhishek Das, Balesh Naik

**Affiliations:** 1 Orthopaedics, Apollo Multispeciality Hospitals, Kolkata, IND

**Keywords:** posterolateral approach to tibia, tumor excision, distal tibial interosseous osteochondroma, bone, osteochondroma

## Abstract

Osteochondromas arising from the interosseous border of the distal tibia are rare, but cases have been reported previously in the literature. In long-standing cases, they can cause a "mass effect" resulting in the deformation of the bones around the ankle joint, mechanical restriction of joint movement, and even degenerative joint disease. Hence, they need to be resected if patients present with such impending complications. Several surgical techniques have been described previously for tumor resection including the anterior approach and the trans-fibular approach, the latter of which required a fibular osteotomy with or without fibular reconstruction. The surgical technique described here utilizes the posterolateral approach to the ankle joint for tumor excision, thus avoiding the need for any osteotomy or fibular reconstruction and reducing the risk of injury to major neurovascular structures. It also reduces the need for long-term immobilization and promotes a faster return to activity.

## Introduction

Osteochondromas are the most common benign bone tumors, accounting for 10% of all primary skeletal tumors, and they present most often in the second decade of life [1,2]. Their growth stops when skeletal maturity is reached. They have been reported to arise from the interosseous border of the distal tibia and cause deformity of the fibula, before skeletal maturity [1]. In long-standing cases, they can cause a "mass effect" wherein the lesion arising from the interosseous border of the distal tibia impinges on the fibula resulting in the plastic deformation of the tibia and fibula, mechanical restriction of ankle joint movement, syndesmotic involvement, angular deformities around the knee or ankle joints, and degenerative joint disease [2,3]. This necessitates a thorough tumor removal while maintaining the stability and motion of the ankle joint [2].

The surgical techniques previously described in the literature for the removal of these tumors include the anterior approach to the distal tibia and the lateral trans-fibular approach [1,2]. The lateral approach requires a fibular osteotomy to gain access to the tumor which necessitates the need for a fibular reconstruction also [2]. These approaches provide access to the tumor, but they also pose a risk of injury to the neurovascular structures and increase the risk of implant-related complications when fibular reconstructions are performed. In our patient, we used the posterolateral approach to the ankle joint for tumor resection without the need for any fibular osteotomy.

## Case presentation

A nine-year-old boy of average build presented to our outpatient department with a complaint of pain, deformity, and a lump over the left ankle for one year. The pain and swelling were insidious in onset and had gradually increased over the past year. There was no history of any difficulty in walking. The remainder of his history was not significant. No other family member had a similar swelling or a history of any bone tumor.

**Figure 1:**
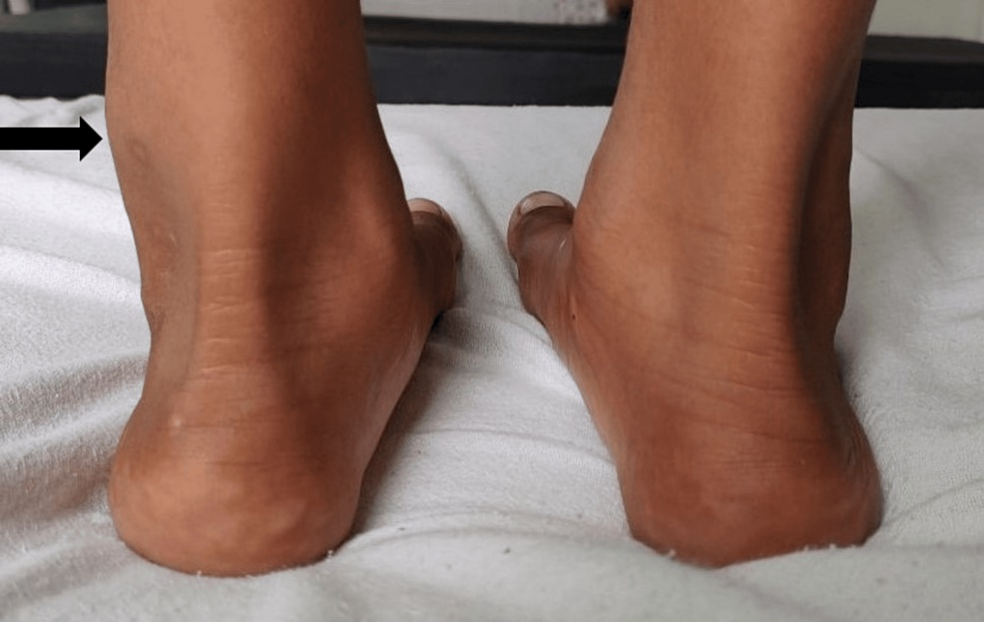
Swelling between the lateral malleolus and the tendoachilles

On physical examination, there was a 3 cm × 3 cm, non-tender, hard swelling, with a smooth surface, palpable between the lateral malleolus and the tendoachilles (Figure 1). Movement at the ankle joint was painless and unrestricted. The neurovascular status was intact. No swelling was palpable in any other joint. The rest of the physical examination was normal.

**Figure 2:**
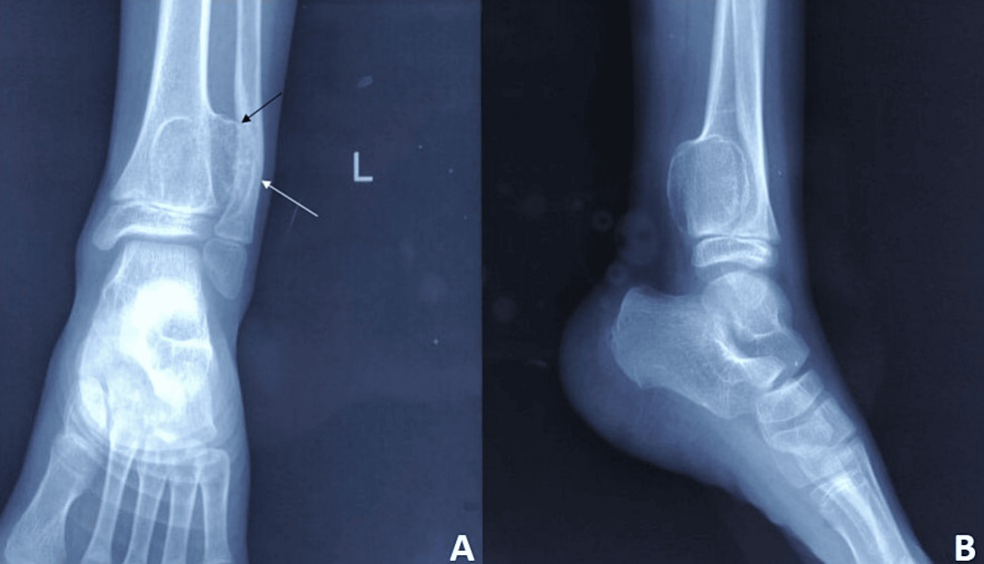
Radiograph showing the mass arising from the interosseous border of the distal tibia A: Anteroposterior view of the ankle; black arrow showing the mass and white arrow showing the fibular deformity. B: Lateral view of the ankle

**Figure 3:**
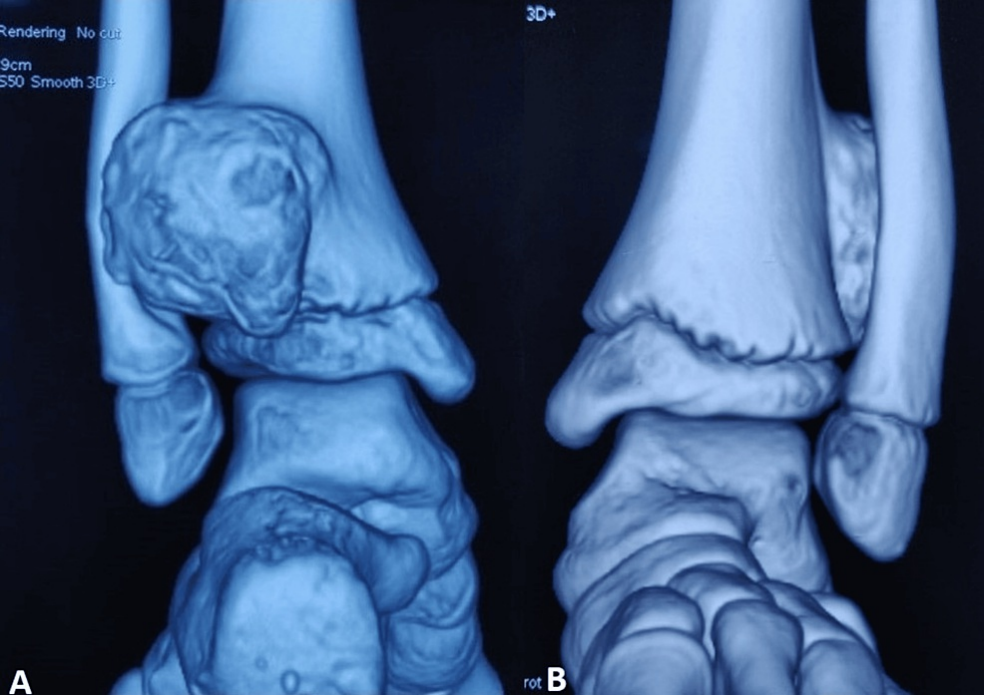
CT scan images A: Mass arising from the posterolateral border of the tibia. B: Gradual remodeling of the fibula CT: computed tomography

Radiographs revealed a large, broad-based, bony outgrowth involving the interosseous border of the distal metaphysis of the left tibia, growing posteriorly and laterally (Figure 2A, 2B). It was partially deforming the left fibula. The cortex and medulla of the swelling were continuous with the bone. The swelling was consistent with an exostosis. A computed tomography (CT) scan of the concerned region revealed an osseous lesion arising from the posterolateral aspect of the distal left tibial metaphysis (Figure 3A). The lesion was abutting the fibula leading to a gradual remodeling of the bone (Figure 3B). A magnetic resonance imaging (MRI) study of the left ankle confirmed the lesion to have an uninterrupted cortico-medullary continuity with the parent bone with a broad-based communication. The lesion measured 3.1 cm in the coronal plane, 2.8 cm in the anteroposterior plane, and 1.8 cm in the transverse plane. The overlying cartilage cap had a maximum thickness of 2 mm. The lesion was seen to cause a compressive effect and remodeling of the distal fibular metaphysis.

After a thorough discussion with the patient's parents, we decided to go ahead with a surgical intervention, to prevent the progression of the fibular deformity. Various surgical approaches were considered, and we decided to excise the mass through the posterolateral approach to the ankle.

The procedure was performed under spinal anesthesia on a standard operating table. The patient was placed in a supine position. A tourniquet was used to provide a bloodless operative field and better visualization. A medium-sized bolster was placed under the ipsilateral hip to keep the leg in slight internal rotation, allowing better leg stability. The ankle was prepped and draped.

**Figure 4:**
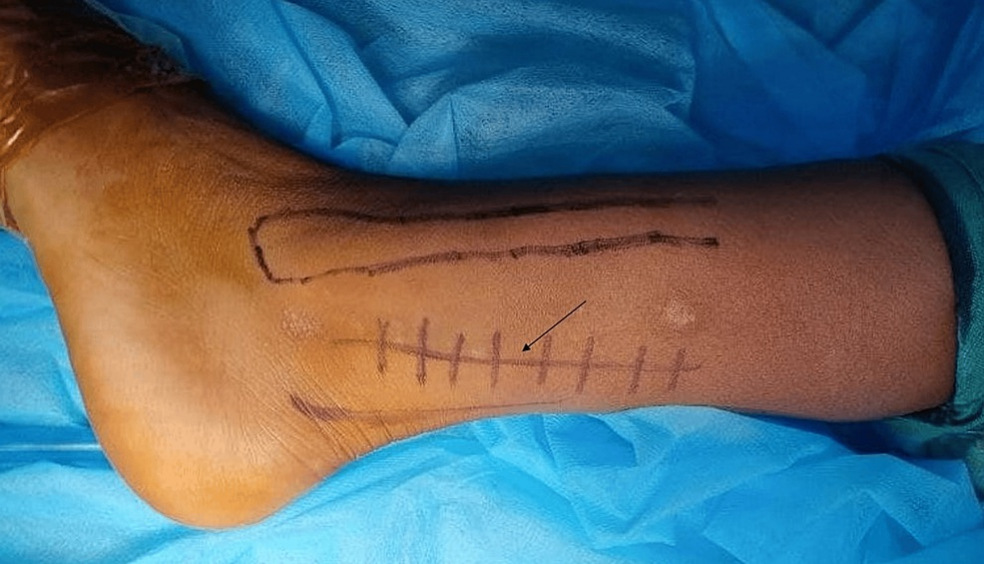
Longitudinal skin incision between the lateral malleolus and the tendoachilles

**Figure 5:**
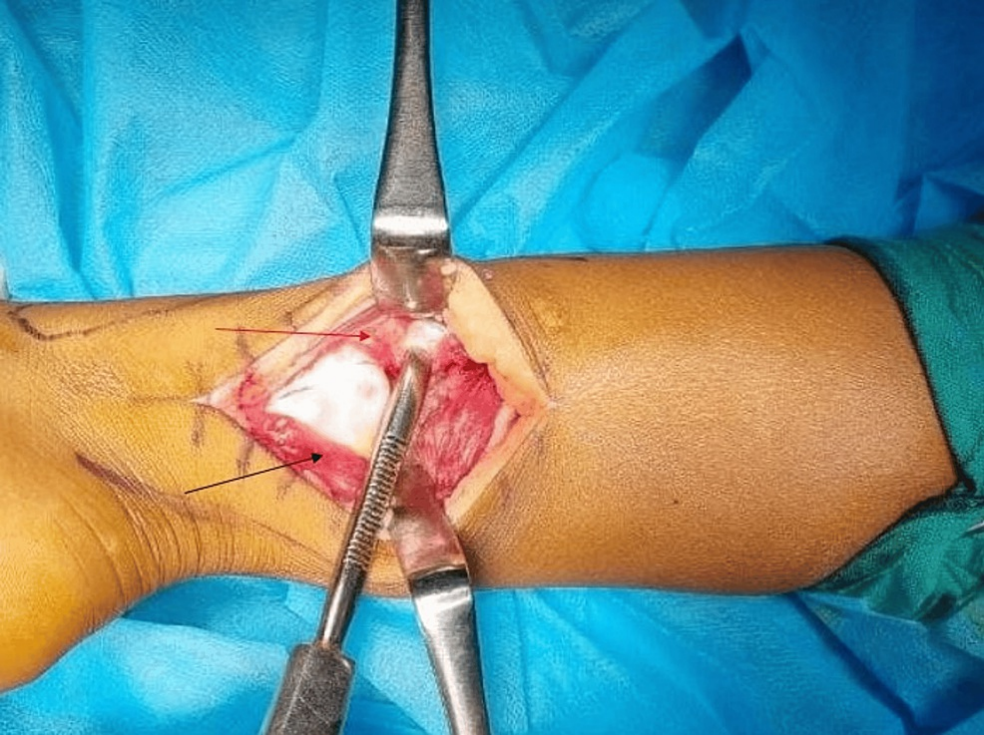
Plane between the peroneus muscles anteriorly and laterally (red arrow) and the flexor hallucis longus posteriorly and medially (black arrow)

**Figure 6:**
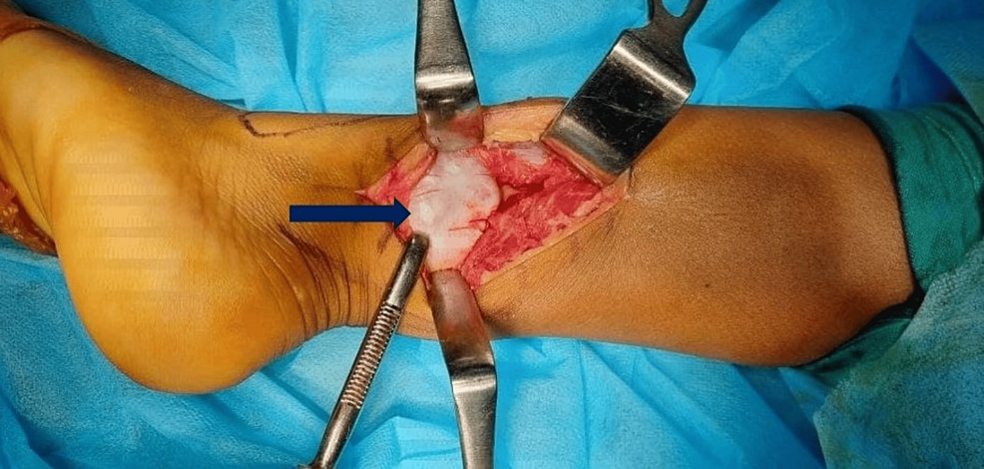
Exposure of the lesion

**Figure 7:**
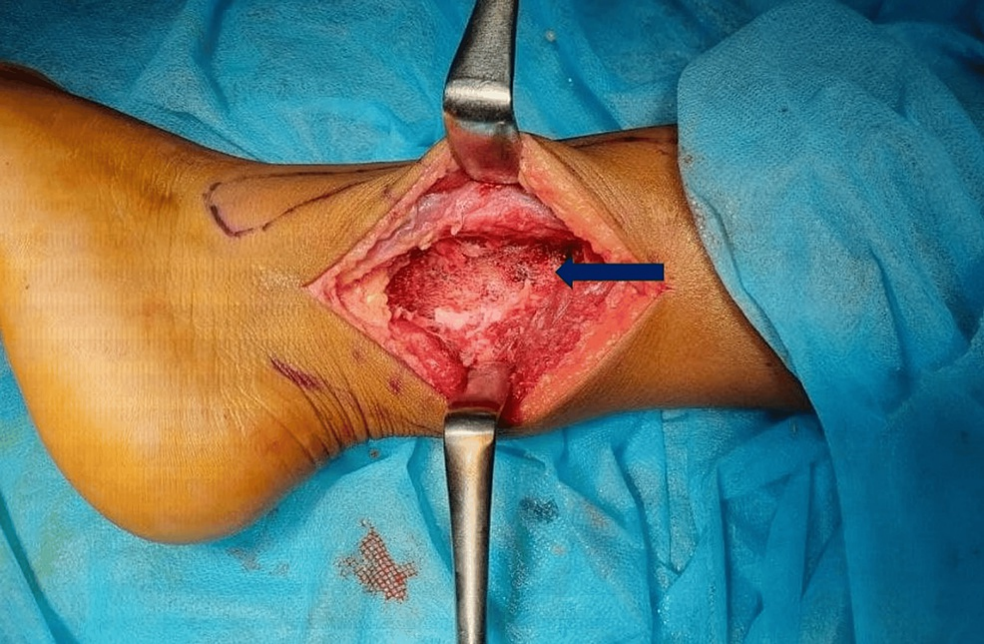
After complete removal of the lesion

**Figure 8:**
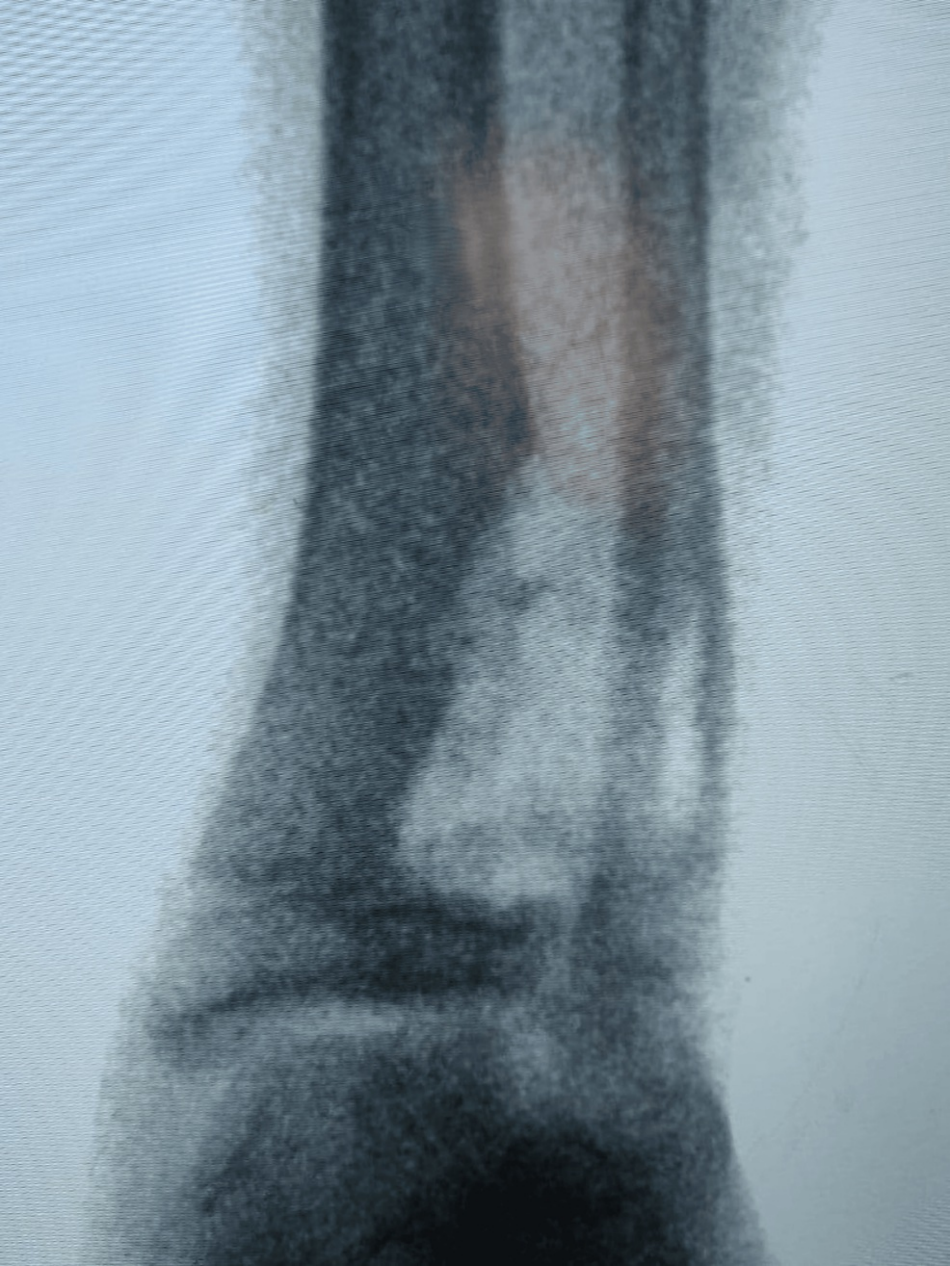
Intra-operative fluoroscopy image showing adequate tumor excision

The tourniquet was inflated after intravenous antibiotic administration. The lateral malleolus was palpated subcutaneously. The tendoachilles was also easily palpable in the distal part of the leg. A longitudinal skin incision was made mid-way between the posterior border of the lateral malleolus and the lateral border of the tendoachilles (Figure 4). The deep fascia of the leg was incised in line with the skin incision. The inter-nervous plane was formed between the peroneus muscles supplied by the superficial peroneal nerve and the flexor hallucis longus muscle supplied by the tibial nerve (Figure 5). The peroneal retinaculum was incised to release the tendons, and the muscles were retracted anteriorly and laterally. The flexor hallucis muscle was then exposed, and the lateral fibers were incised longitudinally. It was then retracted medially to reveal the osteochondroma lesion (Figure 6). After identifying the boundaries of the lesion, the mass was completely resected with an osteotome and a hammer (Figure 7). Adequate resection was confirmed intra-operatively with fluoroscopy (Figure 8). There was no instability of the syndesmosis. After thoroughly washing the wound, it was sutured in layers and a sterile dressing pad was placed.

**Figure 9:**
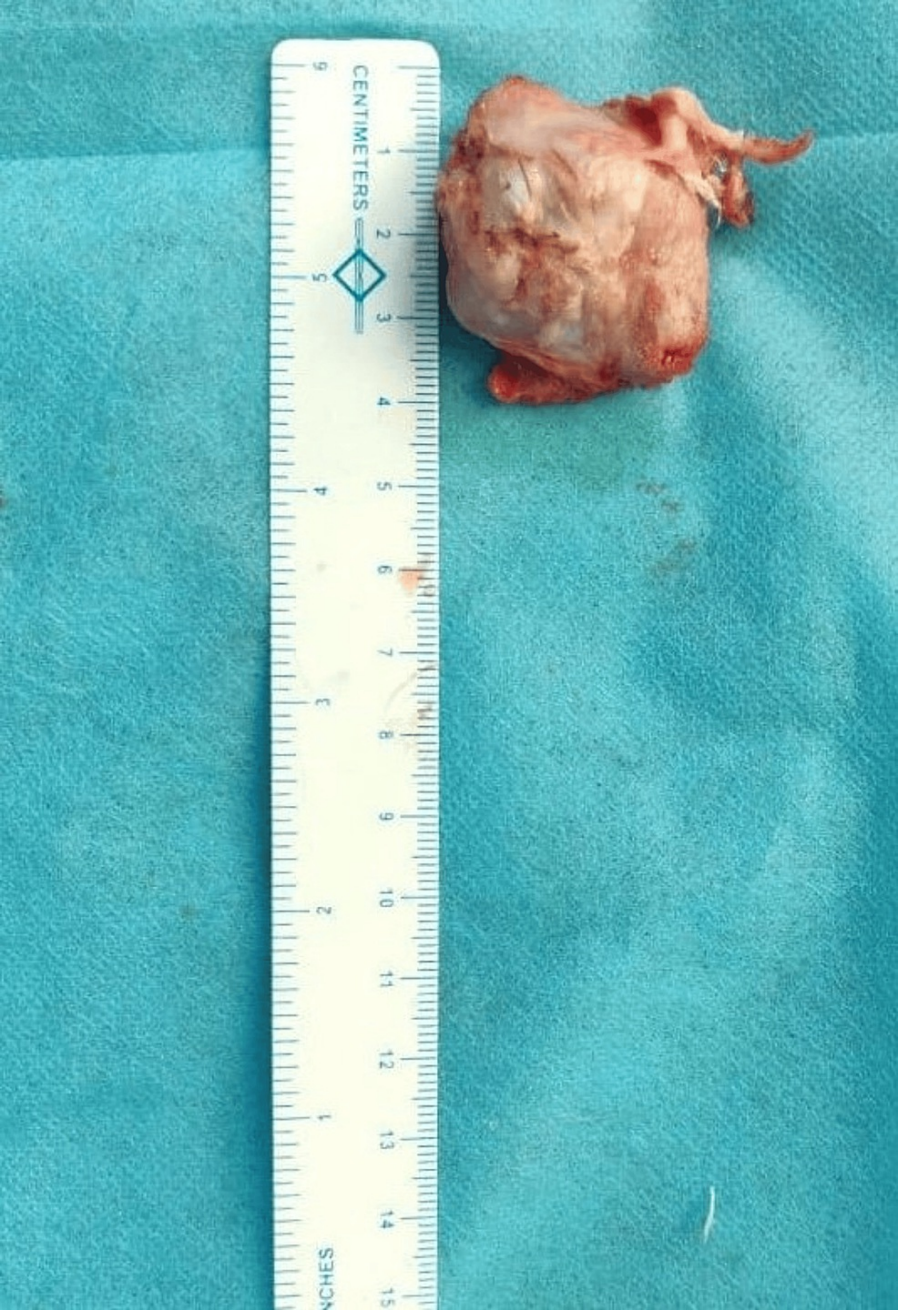
Excised osteochondroma lesion

The excised lesion was approximately 3 cm × 3 cm × 2 cm in dimension (Figure 9). Histopathological examination confirmed the lesion to be an osteochondroma. Range of motion exercises for the ankle were started the next day after surgery. Partial weight bearing was allowed on the fifth post-operative day. Sutures were removed two weeks after the surgery. Full weight bearing was allowed from the third week. The patient had no signs of tumor recurrence at the six-month follow-up visit.

## Discussion

Osteochondroma or osteocartilagenous exostosis is known to be the most common benign bone tumor [4]. Lesions that are asymptomatic or incidentally discovered may be observed and managed conservatively. Resection is reserved for patients who have a lesion that is repeatedly exposed to minor trauma, is symptomatic due to irritation of the surrounding soft tissue, and is causing a cosmetic deformity or affecting the surrounding joints or neurovascular structures and those which have characteristics of malignant transformation [3,5].

A variety of surgical approaches have been described for tumor removal from the distal tibia interosseous border. An anterior approach has been described previously by various other authors [1,5-7]. This approach provided a more stable ankle joint post-operatively as the fibula was preserved and the fibula also underwent a gradual remodeling [7]. However, there are certain disadvantages associated with this procedure. Lesions in the posterior or posterolateral aspect of the distal tibia may be difficult to access via this approach, and it also carries a risk of damage to neurovascular bundles, as they reside in close proximity to the incision used in this approach [8].

Durak et al. performed a distal fibular resection for treating a fibular osteochondroma [9]. This approach provided easier and better access to posteriorly located distal tibia osteochondroma and reduced the risk of damage to the neurovascular structures. The drawback of this approach was that complete fibulectomy without any reconstructive procedure compromised the stability of the ankle joint [8]. Therefore, this procedure is considered to be obsolete [10].

To overcome this, Gupte et al. performed a resection of distal tibia osteochondroma via a direct lateral approach. They performed a fibular osteotomy, followed by repair of the fibula with semi-tubular plates and a diastasis screw [8]. Similar techniques of distal fibular osteotomy and rotational fibular osteotomy have been described by other authors [11,12]. These techniques eliminate the risk of damage to the anterior neurovascular bundles and extensor tendons, provide easy access to posteriorly located tumors, and also maintain the stability of the ankle joint. However, they also carry a theoretical risk of damage to the lateral neurovascular structures, which include the sural nerve, its accompanying artery, as well as the perforating peroneal artery [8]. The patients are not allowed to bear weight on the operated limb for four to six weeks to allow healing of the osteotomy site, which significantly hampers mobility and delays the return to activity. A well-known complication with this procedure is the non-union of the fibular osteotomy which necessitated a revision surgery [11,12]. Distal tibiofibular synostosis was also reported by Frick et al. when osteotomies of the tibia and fibula were performed simultaneously in children [13].

Thakur et al. performed an excision of a similar mass via the trans-fibular approach, where the fibula was removed, and reconstruction was done by Sofield's method using a radius square nail. But in this case, the nail had migrated, and there was a need for a second surgery for implant removal [2].

The posterolateral approach is a safe and well-known approach, and its use for the excision of tumors arising in this area can be considered a viable option. This approach has been reported previously with no known complications [14,15]. Liu et al. also used a fibular plate and an elastic loop plate fixation for the distal tibiofibular syndesmosis following tumor excision due to a pre-existing syndesmosis injury [15]. The fibula is known to undergo gradual remodeling in younger patients with an open physes, also to some extent after the closure of distal fibular physes [7,14].

## Conclusions

The posterolateral approach used in our patient does not carry the risk of injury to the anterior neurovascular bundles or extensor tendons and provides easy access to posteriorly located tumors. As the ankle joint is maintained, there is no risk of ankle instability. The risk of tibiofibular synostosis is eliminated, and there is no need for a second surgery as it does not involve any osteotomy or reconstruction of the fibula.
